# Beauvericin production by the Lepidoptera pathogenic fungus *Isaria tenuipes*: Analysis of natural specimens, synnemata from cultivation, and mycelia from liquid-media fermentation

**DOI:** 10.1007/s13659-011-0038-0

**Published:** 2011-12-07

**Authors:** Sumalee Supothina, Urarat Srisanoh, Sutichai Nithithanasilp, Kanoksri Tasanathai, J. Jennifer Luangsa-Ard, Chun-Ru Li, Masahiko Isaka

**Affiliations:** 1 National Center for Genetic Engineering and Biotechnology (BIOTEC), 113 Thailand Science Park, Phaholyothin Road, Klong Luang, Pathumthani, 12120 Thailand; 2 Anhui Provincial Key Laboratory for Microbial Control, Anhui Agricultural University, Hefei, 230036 Anhui, China

**Keywords:** beauvericin, *Isaria tenuipes*, chemotaxonomy, toxin

## Abstract

Beauvericin was analyzed in three forms of the Lepidoptra pathogenic fungus *Isaria tenuipes* (4 isolates): (a) natural specimen, (b) cultivated synnemata on rice media, and (c) mycelia from fermentation in liquid media. Beauvericin was detected in very low amounts in all tested natural specimens. Synnemata on rice contained much higher concentrations of beauvericin than the corresponding natural materials, although the concentrations were lower than mycelia from liquid fermentation. The results casted a caution that beauvericin concentration should be carefully checked, as a possible toxic constituent, upon mass production of a selected strain of *Isaria tenuipes* for health food purposes.

## Introduction

Beauvericin is a cyclohexadepsipeptide fungal metabolite possessing a broad range of biological activities such as insecticidal, anthermintic, antibacterial, antifungal, anti-plasmodial, antimycobacterial, and anticancer activities.[1] Beauvericin and related fungal cyclooligomer depsipeptides, enniatins, were also reported as inhibitors of acyl-CoA: cholesterol acyltransferase (ACAT)[2] and the human immuno-deficiency virus type-1 integrase.[3] Beauvericin was first described from an entomopathogenic fungus *Beauveria bassiana* on the basis of its toxicity to brine shrimp.[4] In addition to a number of reports from this species and other *Beauveria* species, this compound has also been isolated from several other entomopathogenic fungi such as *Isaria tenuipes* (formerly *Paecilomyces tenuipes*, also known as *Isaria japonica*),[5–6] *Isaria fumosorosea* (formerly *Paecilomyces fumosoroseus*),[7] and *Cordyceps cicadae*.[8] All these species belong to the family *Cordycipitaceae* in the Hypocreales (Ascomycota).[9] *Fusarium* species infecting maize, rice, and wheat are also known as beauvericin producers.[10–12] While beauvericin is produced as the sole cyclohexadepsipeptide metabolite by the former entomopathogenic fungi, it is often co-produced as a minor constituent with the structurally related enniatins by *Fusarium* species.[13–16]

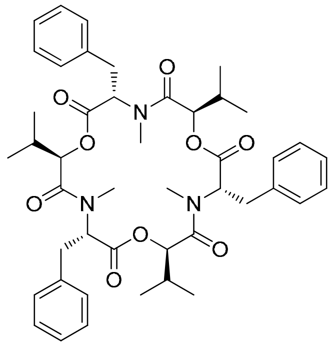

beauvericin

Recently we reported that *Isaria tenuipes*, a specific pathogen of Lepidoptera pupa or larva, and several phyloge-netically closely related species constantly produce beauver-icin when fermented in potato dextrose broth (PDB).[17] *Isaria tenuipes* has been used as folk medicines or health food, for good health, in East Asian countries, Japan, China, and South Korea. Extracts from mass cultivated synnemata on insect larvae (e.g. silkworm) or solid media are used for commercial products. As for low molecular weight metabolites from this fungus, a group of trichothecanes, paecilomycine A,[18] tenuipesines A and B,[19] and spirotenuipesines A–C,[20] have been isolated from the synnemata grown on silkworms, where-

*To whom correspondence should be addressed. E-mail: isaka@biotec.or.th as a pseudodipeptide, hanasanagin (3,4-diguanidino-butanoyl-DOPA) was isolated by other group.[21] Our chemotaxonomy results posted a question of a toxicity risk to humans due to the presence of beauvericin in synnemata, in the particular case of administration in high frequency and dosage. Indeed, beauvericin is known to be cytotoxic to human cells, and it has been considered as one of the problem toxins produced by *Fusarium* species in crops.[1,22] In addition, we were also interested in whether or not beauvericin is present in natural specimens of *Isaria tenuipes*. We therefore undertook quantitative analysis of beauvericin in three different forms of *Isaria tenuipes* from the same natural source: (a) natural specimen (synnemata and insect cadaver), (b) synnemata cultivated on rice, and (c) mycelia of liquid-media fermentation broth.

## Results and Discussion

Four natural specimens of *Isaria tenuipes* synnemata attached to Lepidoptera pupae were collected in July 2008–February 2009 (Table 1). Corresponding living cultures were deposited in the BIOTEC Culture Collection (BCC), and they were used for growing synnemata on rice (Figure 1) and incubation of mycelia in a liquid medium. Beauvericin in extracts from cultivated synnemata and fermentation mycelia was analyzed by $^1$H NMR (400 MHz, $CDCl_3$) using *N*,*N*-dimethylformamide (DMF) as an internal standard (Figure 2). Synnemata of natural specimens were picked from the insect cadaver, and they were separately extracted in MeOH. Weight of the synnemata of each natural specimen ranged 14–35 mg. Weight of the insect cadaver (Lepidoptera pupa) ranged 13–41 mg. Because of the low material quantity and the low beauvericin concentration in each material, HPLC-MS was employed for the analysis. The quantification of beauvericin was achieved by HPLC chromatogram (UV 210 nm) using ethyl 4-phenylbenzoate (0.01 % weight of synnemata or insect cadaver) as an internal standard. The presence of beauvericin was confirmed by ESIMS (positive ion mode).

The results of beauvericin analysis are summarized in Table 1. Most notably, beauvericin was detected in natural synnemata and insect cadavers of all tested materials. The compositions of beauvericin in insect cadavers were relatively higher than that in the corresponding synnemata. Cultivated synnemata on rice also contained beauvericin with much higher compositions than the corresponding natural specimens. Most effective beauvericin production was performed by liquid fermentation. The beauvericin concentrations in freeze-dried mycelia were higher than those in cultivated synnemata.

This is the first report of the detection of beauvericin in natural specimens of *Isaria tenuipes*. In all natural materials, the beauvericin concentrations were very low and showed differences between the isolates. This difference may related in part to the age of the material, since beauvericin should degrade slowly under natural conditions like any other metabolites. To our knowledge, beauvericin isolation from cultivated synnemata of *Isaria tenuipes* (or *Paecilomyces tenuipes*, *Isaria japonica*) has also not been reported. Synnemata of *Isaria tenuipes* on rice contained considerably high concentrations of beauvericin, although lower than liquid fermentation.

*Cordyceps cicadae*, infecting on larvae of *Cicada flammata*, is another Chinese folk medicine. In ancient China, it has been reported to improve vision and other eye problems.[23] There was a report of the isolation of beauvericin from insect cadaver portion of *Cordyceps cicadae*, commercial folk medicine material.[8] However, the yields of beauvericin and other compounds from the huge material (9.7 kg) was not presented. In our chemotaxonomic study on *Isaria*,[17] a Thai strain of *Isaria cicadae* (BCC 13914) and a *Cordyceps* state (teleomorph) of this species (*Cordyceps cicadae* BCC 15603) were involved in the "beauvericin clade" together with *Isaria tenuipes* in the phylogenetic relations. These two strains produced beauvericin by liquid fermentation, but the respective productivities of 0.6 and 0.5 mg/L in PDB were much lower when compared to *Isaria tenuipes* strains under the same fermentation conditions.[17]

Mass cultivations of *Isaria tenuipes* synnemata for commercial purposes have been achieved by infecting this fungus on insect larvae (commonly silkworm) or growing on solid media (e.g., rice). The synnemata cultivation condition employed in the present study is limited to one of various methods, which should be the primary factor to control the efficacy of beauvericin production. For comparison, we also examined growing *Isaria tenuipes* synnemata by infecting

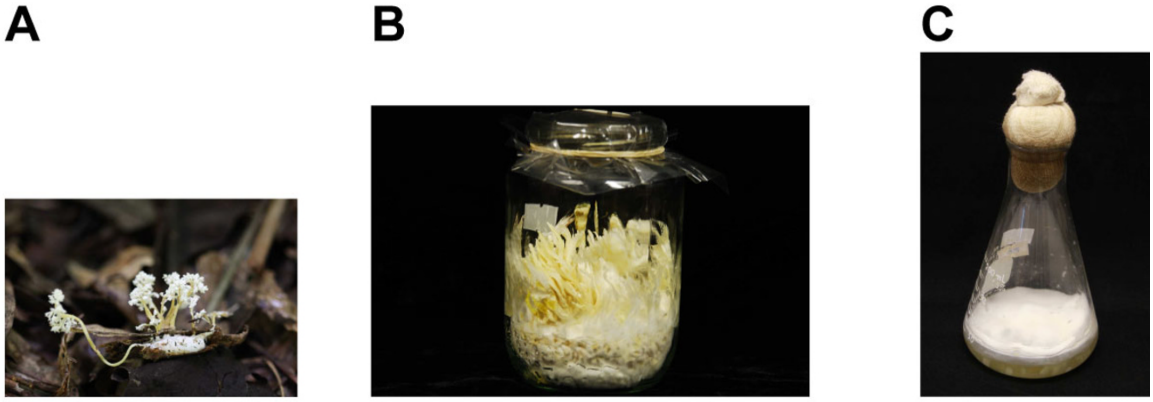

**Figure 1.** Photos of *Isaria tenuipes* BCC 31640 in three different forms. (**A**) Natural specimen on a Lepidoptera pupa. (**B**) Synnemata cultivation on rice. (**C**) Fermentation in minimal salt medium (MM).

**Table 1. List of *Isaria tenuipes* isolates and their beauvericin productivity.**

| code | location, province; date of collection[a] | natural specimen | | cultivation | fermentation |
|---|---|---|---|---|---|
| | | synnemata | insect cadaver | synnemata | mycelia |
| | | mg/g | mg/g | mg/g | mg/g (mg/L) |
| BCC 31640 | Huay Nam Dang National Park, Chiang Mai; July 5, 2008 | 0.036 | 0.058 | 0.915 | 14.1 (87.2) |
| BCC 33299 | Khao Yai National Park, Nakhon Nayok; November 4, 2008 | 0.038 | 0.030 | 0.689 | 18.1 (61.1) |
| BCC 35849 | Khao Yai National Park, Nakhon Nayok; February 5, 2009 | 0.0017 | 0.011 | 0.347 | 0.49 (4.0) |
| BCC 35850 | Khao Luang National Park, Nakhon Si Thammarat; February 17, 2009 | 0.0056 | 0.076 | 0.637 | 30.3 (100.2) |

[a]All these natural specimens were collected on Lepidoptera pupae.

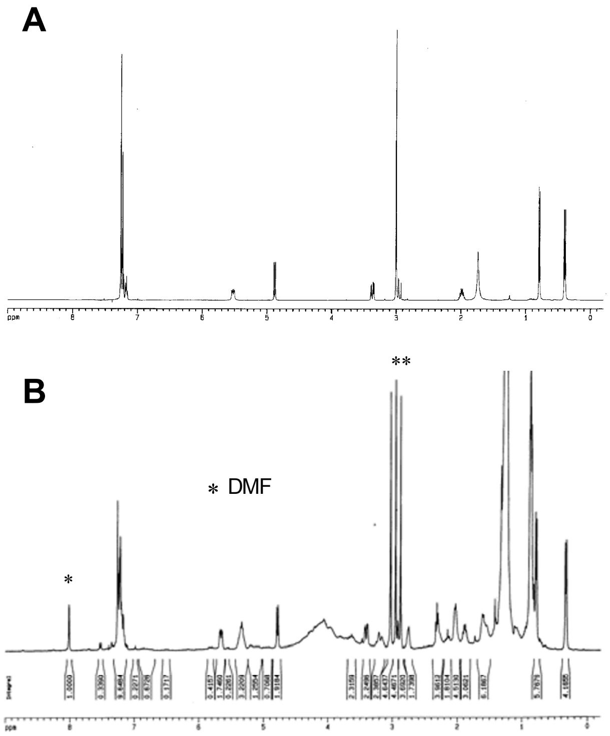

**Figure 2.** $^1H$ NMR spectra (400 MHz, $CDCl_3$). (**A**) beauvericin (standard sample). (**B**) Extract from synnemata of *Isaria tenuipes* BCC 33299 on rice with DMF (10 $\mu$mol, internal standard).

larvae of *Spodoptera exigua*. However, the conditions that succeeded in growing using a Chinese strain of *Isaria tenuipes*[24] have not been applicable for Thai strains. The efficacy of beauvericin production will also differ between the strains of *Isaria tenuipes*, and there are also strains that do not produce beauvericin.[17] Nonetheless, the present results cast a caution that beauvericin concentration should be carefully checked upon mass production of a selected strain of *Isaria tenuipes* for health food purposes.

## Experimental Section

**Fungal Material.** Four natural specimens of *Isaria tenuipes* were collected on Lepidoptera–pupa from three locations during July 2008–February 2009 by one of the authors (K. Tasanathai). Living cultures were deposited in the BIOTEC Culture Collection with the strain codes as shown in Table 1. Then, the natural specimens were subjected to methanolic extraction within 2 weeks of sample collection.

**Extraction of Natural Specimens.** The natural specimen corresponding to strain BCC 31640 was dried in vacuo for 16 h, and separated to synnemata (35 mg) and insect cadaver (41 mg). The synnemata were extracted with MeOH (5 mL, 2 days) and filtered. The filtrate was defatted by partitioning with hexanes (5 mL). The MeOH layer was evaporated, and the residue was dissolved in EtOAc (5 mL), washed with $H_2O$ (2 mL), and evaporated. The residue was dissolved in 70 $\mu$L MeCN containing 0.1 mg/mL of ethyl 4-phenylbenzoate (internal standard; 7.0 $\mu$g, 0.02% weight of synnemata) and used for LC-MS analysis. The insect cadaver was also extracted in the same manner and the extract was dissolved in 82 $\mu$L of 0.1 mg/mL ethyl 4-phenylbenzoate solution in MeCN (8.2 $\mu$g, 0.02% weight of insect cadaver). In the same fashion, extraction samples of other natural specimens were prepared.

**LC-MS Analysis of Extracts from Natural Specimens.** LC-MS analysis was performed using Bruker MicrOTOF mass spectrometer connected to Aglient 1200 Series HPLC system. A 3–10 $\mu$L portion was injected for HPLC: column, Waters XBridge C18, 2.1 × 50 mm, 3.5 $\mu$m; mobile phase, MeCN/$H_2O$, gradient from 0:100 to 100:0 over 10 min, then 100:0; flow rate, 0.3 mL/min; detection, UV 210 nm. ESITOF mass trace (positive ion mode) was obtained for identification of beauvericin and internal standard. The HPLC retention times of beauvericin and ethyl 4-phenylbenzoate were 12.3 and 10.9 min, respectively. The beauvericin concentration was calculated from its peak area in the chromatogram (UV 210 nm), relative to the internal standard. The limit of quantification was 0.6 $\mu$g/g. The limit of detection (ESIMS), based on the point having signal-to-noise (S/N) ratio of 10, was less than 0.1 $\mu$g/g.

**Cultivation of Synnemata of *Isaria tenuipes* on a Rice Media and Extraction.** The protocol established at the Anhui Agricultural University[24] was applied for growing synnemata of *Isaria tenuipes* Thai strains. Each strain of *Isaria tenuipes* was maintained on potato dextrose agar (PDA) at 25 °C, the agar was cut into plugs and inoculated in a 250 mL Erlenmeyer flask containing 50 mL of potato dextrose broth (PDB; potato starch 4.0 g/L, glucose 20 g/L, in distilled water), and it was incubated on a rotary shaker (200 rpm) at 25 °C for 7–10 days. A portion (4 mL, 10% v/w) of this seed culture was transferred into a rice medium (rice 40 g soaked in 60 mL of 2% (w/v) silkworm powder liquid for 2 h, then autoclaved at 120 °C for 30 min) in a 400 mL wide mouth glass jar. The inoculated medium was incubated at 25 °C in darkness until mycelia had grown (7–10 days). The bottle was then placed in an ectothermic chamber maintained at 22 ºC, 90 % relative humidity and 1,000 lux of light density, and cultivated for 14–21 days. Synnemata were picked, air-dried and further dried overnight in vacuo, and weighed (0.94–7.50 g). The synnemata were extracted with MeOH (80 mL, 2 days) and filtered. The filtrate was added with $H_2O$ (20 mL) and defatted by partitioning with hexanes (20 mL). The aqueous MeOH layer was evaporated, and the residual aqueous solution was extracted with EtOAc (3 × 30 mL). The combined organic layer was concentrated under reduced pressure to obtain the extract (4.8–69.4 mg). The extract was analyzed by $^1$H NMR (400 MHz, $CDCl_3$) to determine beauvericin amount (internal standard: DMF, 10 $\mu$mol) and to obtain information of other metabolites.[17]

**Fermentation of *Isaria tenuipes* in Liquid Media, and Extraction.** Each strain of *Isaria tenuipes* was maintained on PDA at 25 °C, the agar was cut into plugs and inoculated in 2 × 250 mL Erlenmeyer flasks each containing 25 mL of minimal salt medium (MM; glucose 20 g/L, yeast extract 1.0 g/L, $NH_4NO_3$ 3.0 g/L, $KH_2PO_4$ 0.50 g/L, $MgSO_4 \cdot 7H_2O$ 0.50 g/L, $CaCl_2$ 0.50 g/L, in distilled water), and it was statically incubated at 25 °C for 40–120 days, until reaching a reasonably high mass with a floating thick mycelia fully covering the surface of the media. The cultures were filtered, and the combined mycelia (residue) was washed well with distilled water, and then freeze-dried. After weighing the dried mycelium (160–270 mg), it was extracted by the similar procedure as described above for cultivated synnemata, and the extract (4–59 mg) was analyzed by $^1$H NMR (400 MHz, $CDCl_3$; internal standard, DMF 10 $\mu$mol).

## Acknowledgments

Financial support from the National Center for Genetic Engineering and Biotechnology (BIOTEC) is gratefully acknowledged.

**Open Access** This article is distributed under the terms of the Creative Commons Attribution License which permits any use, distribution, and reproduction in any medium, provided the original author(s) and source are credited.
